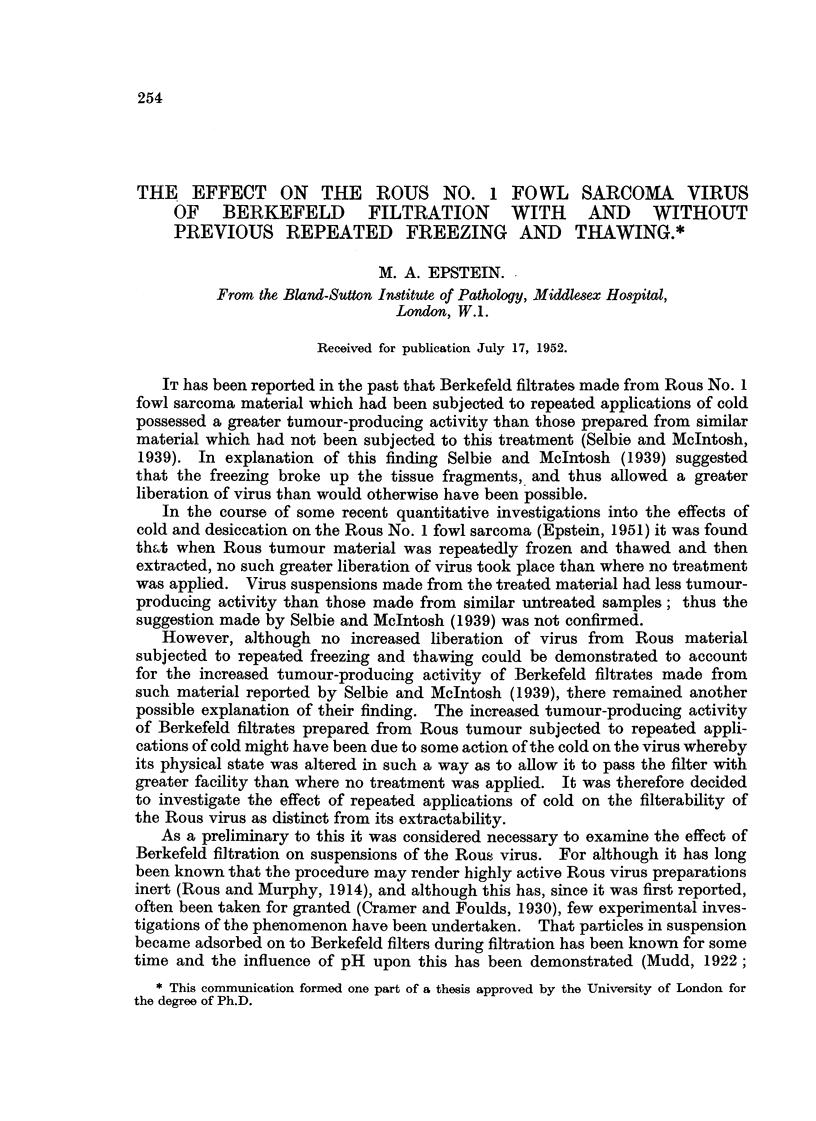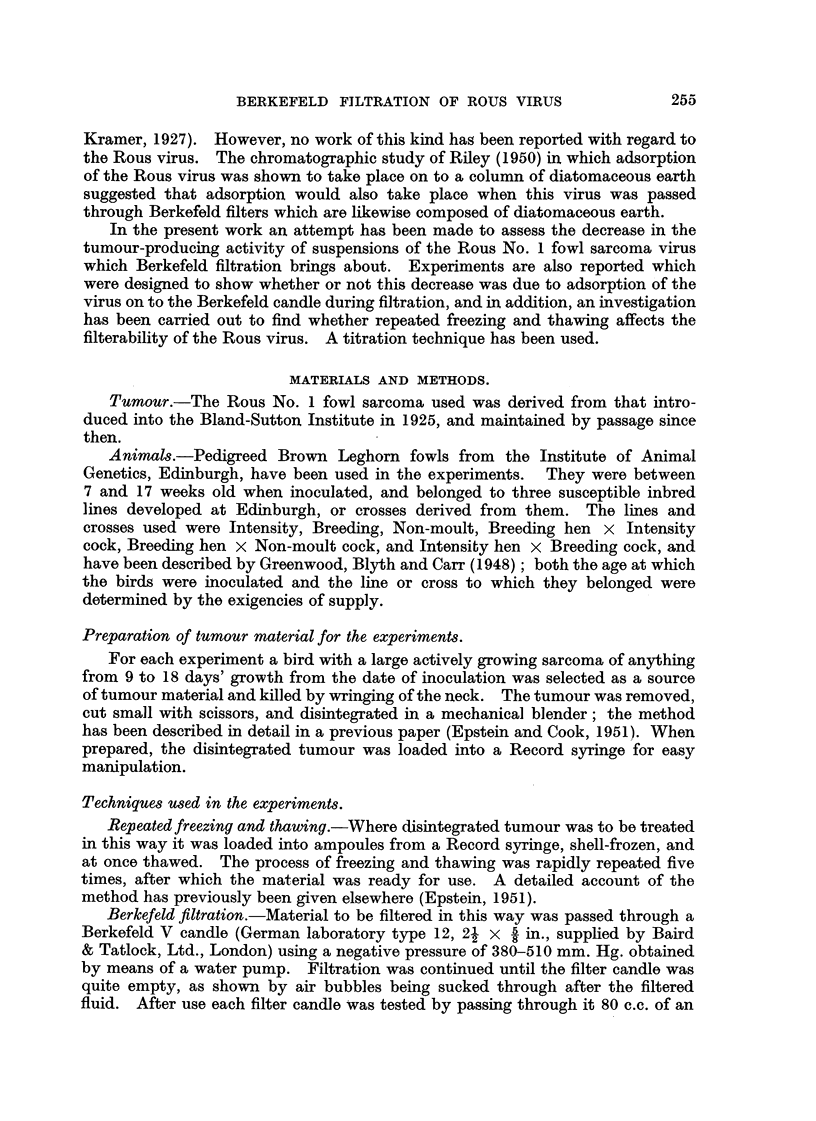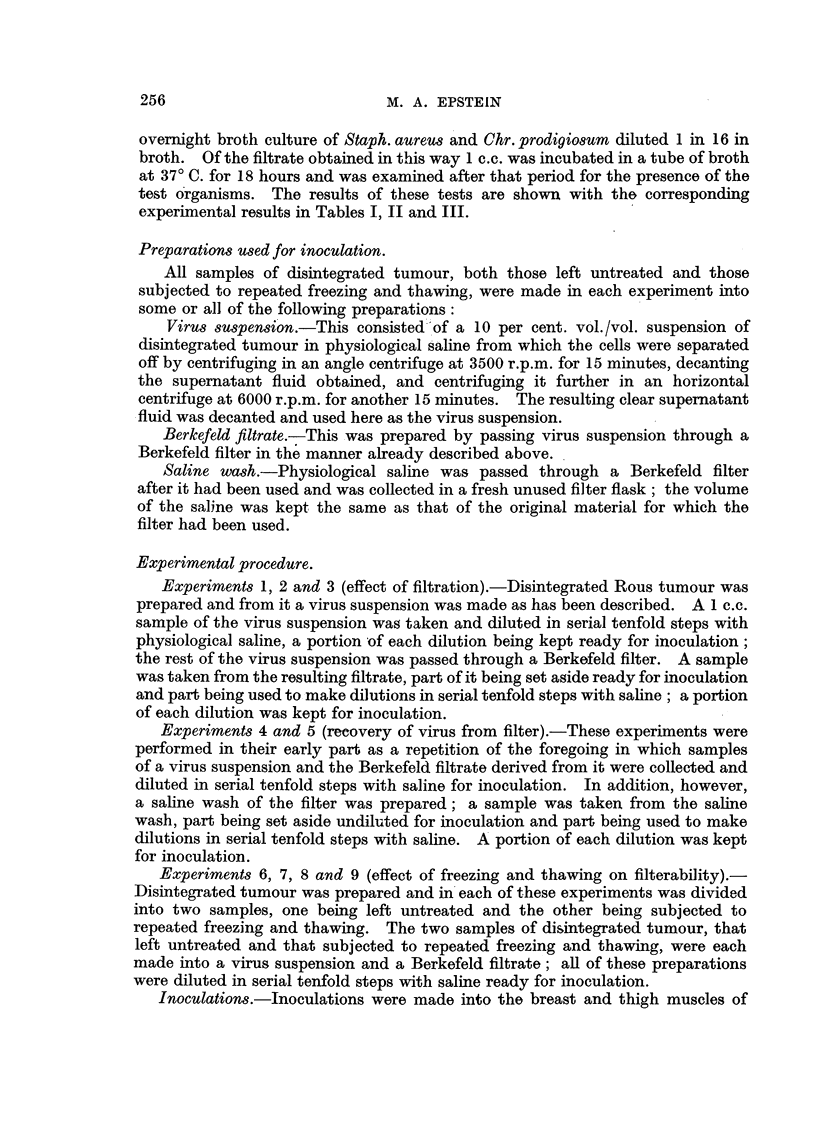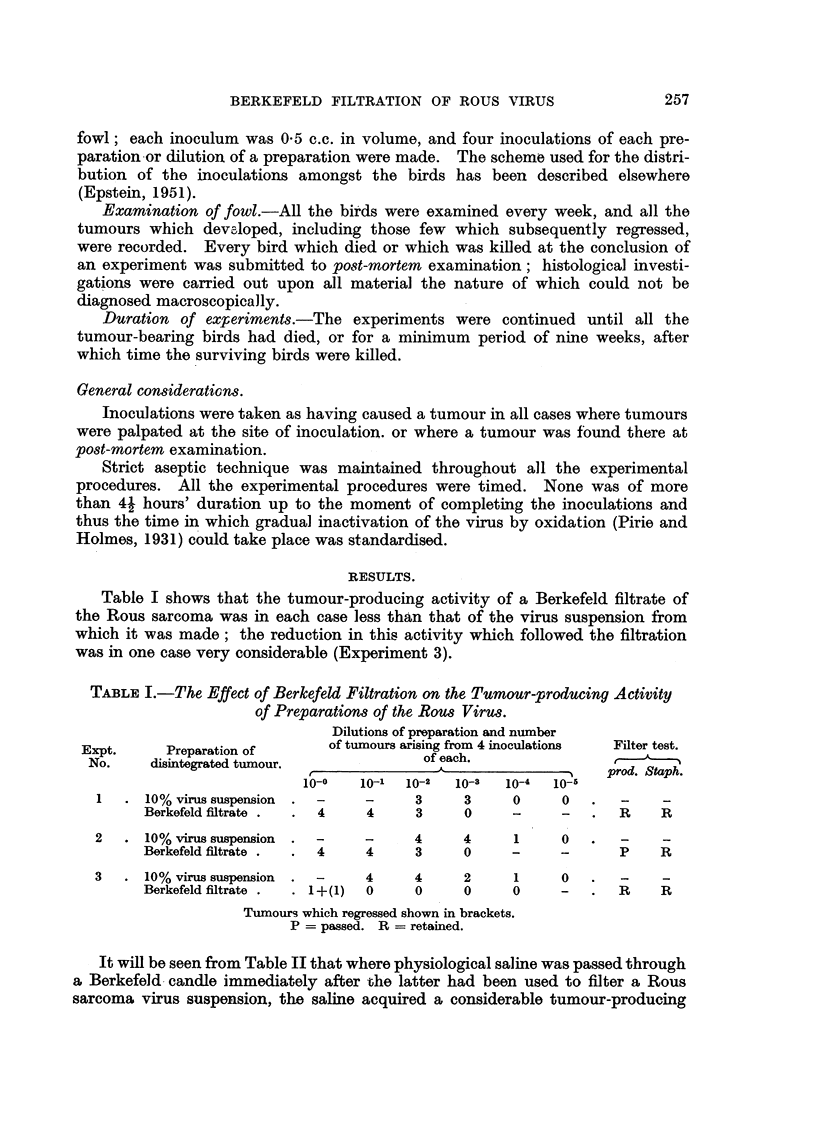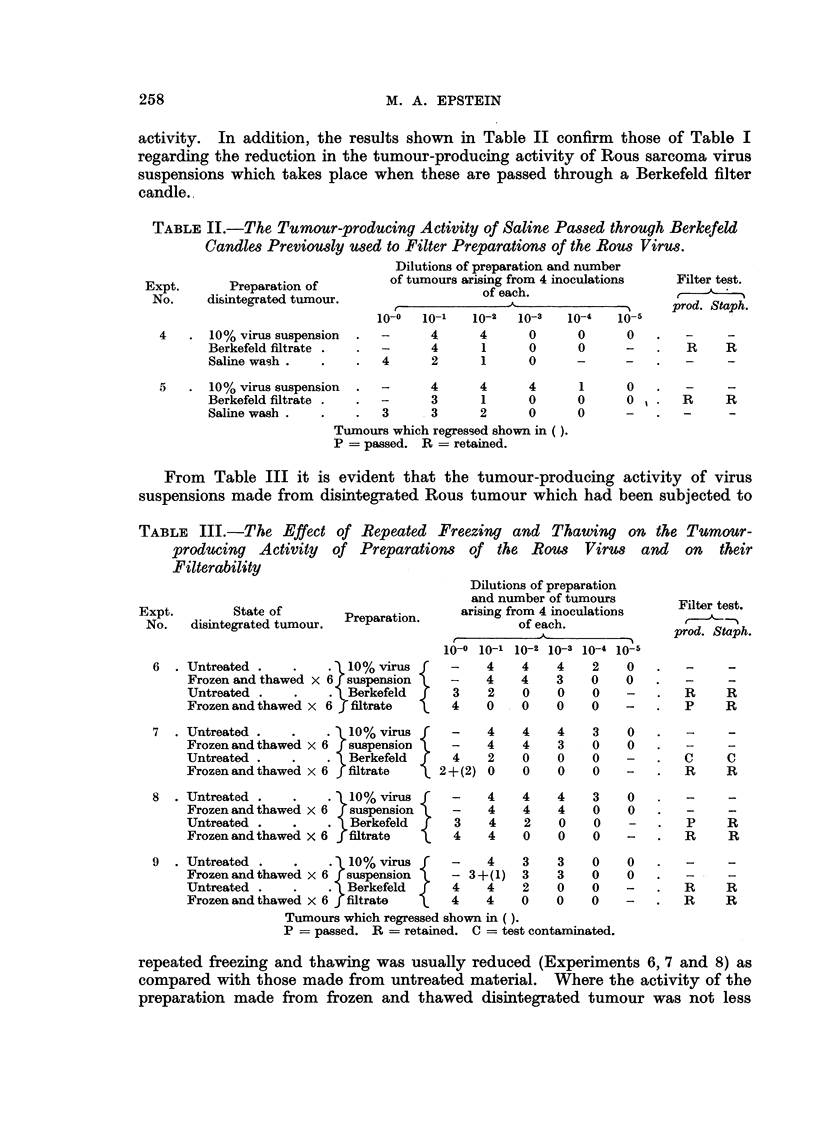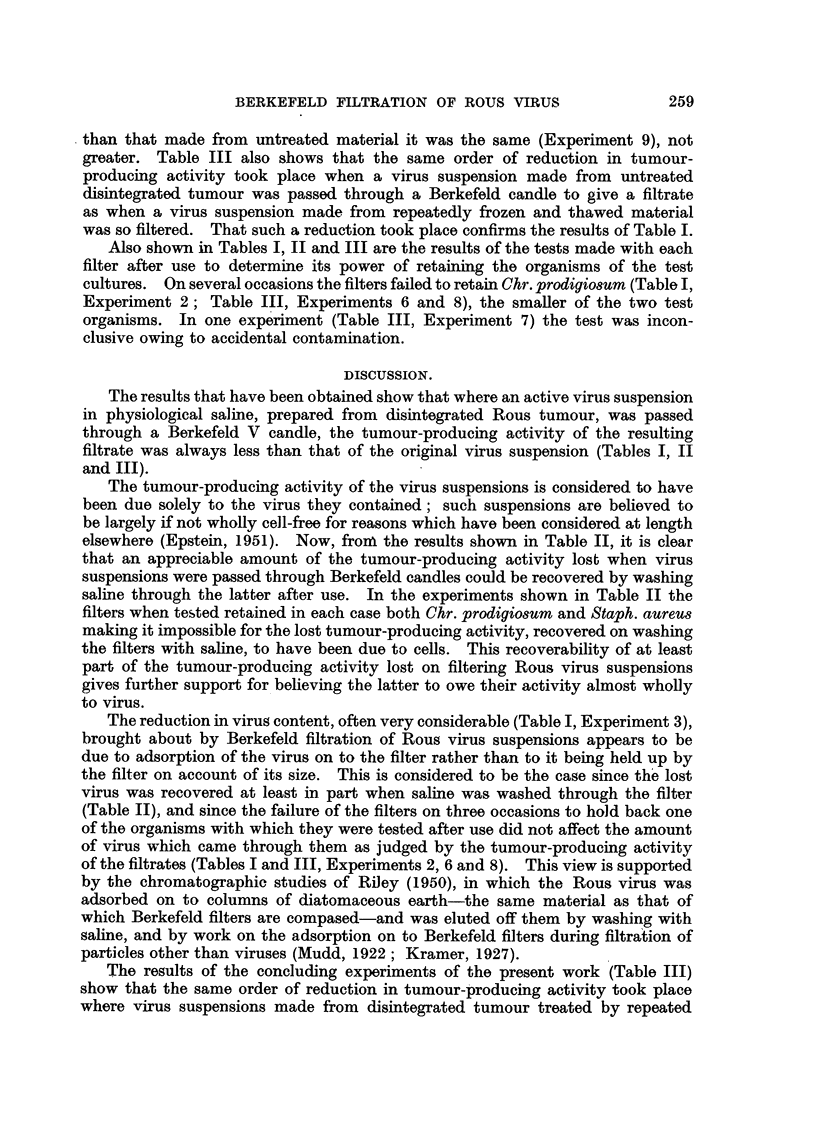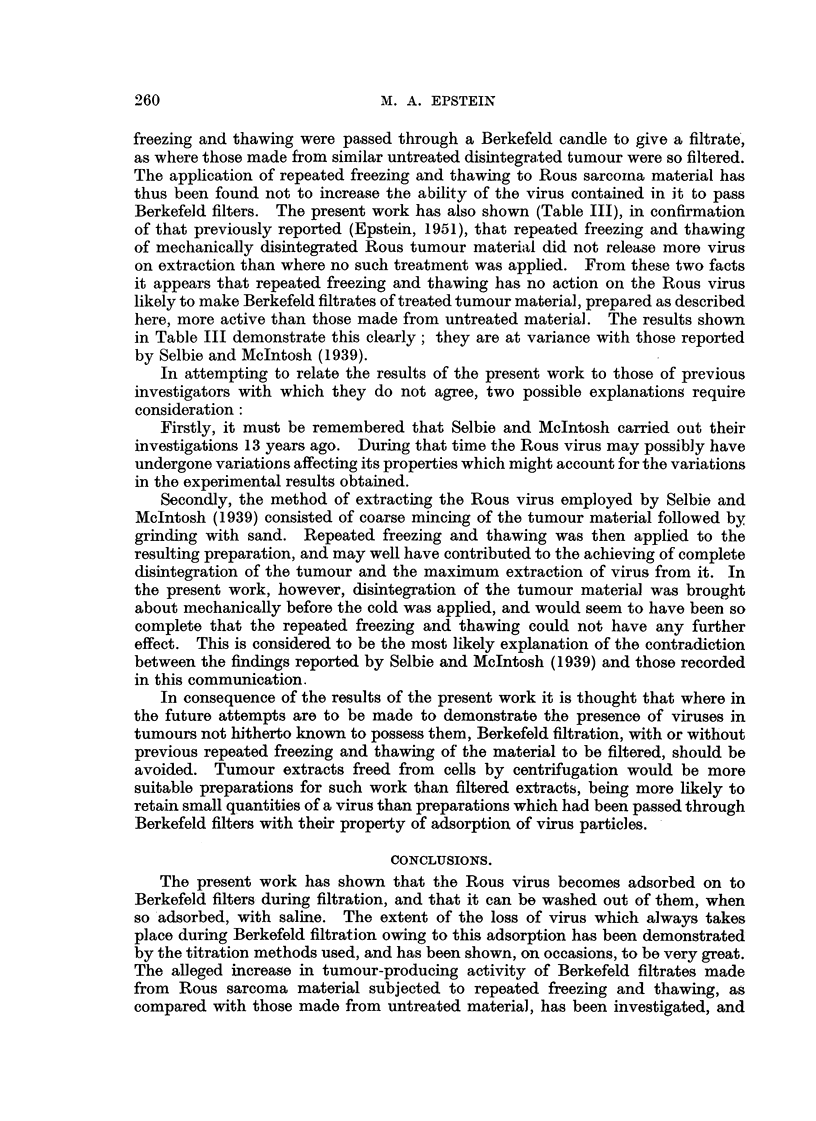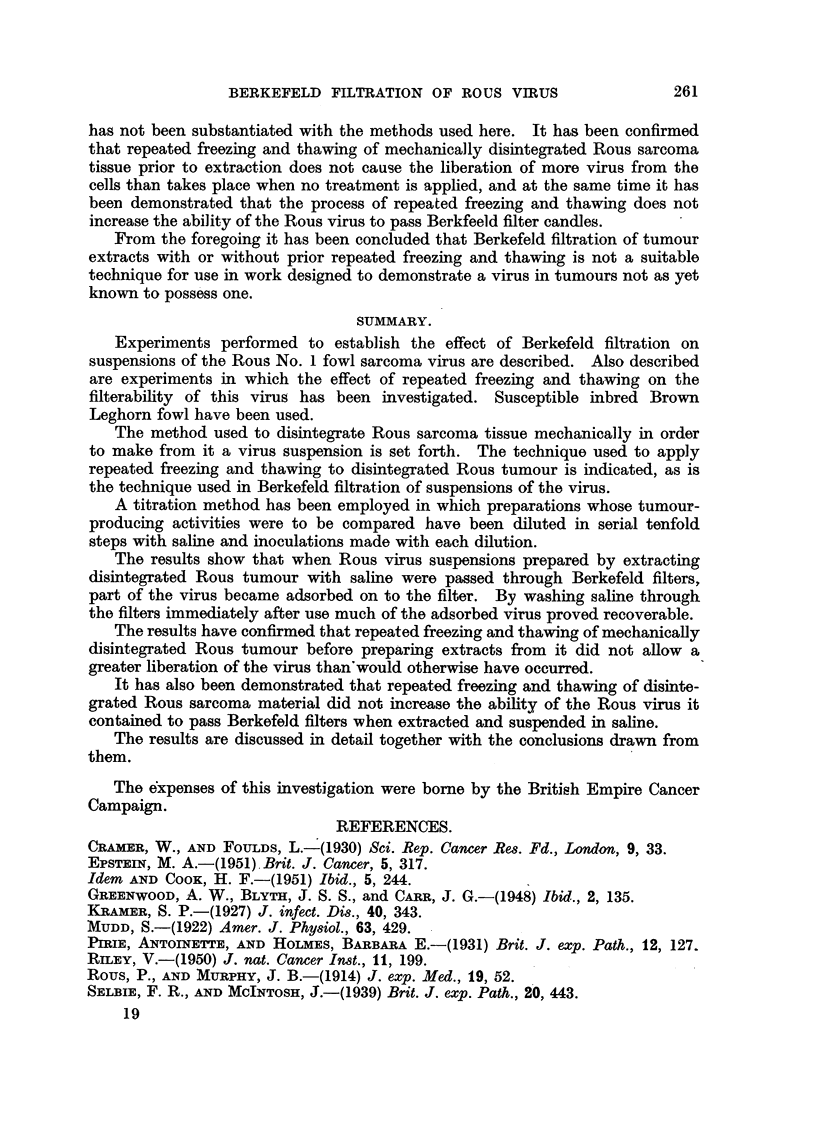# The Effect on the Rous No. 1 Fowl Sarcoma Virus of Berkefeld Filtration with and without Previous Repeated Freezing and Thawing*

**DOI:** 10.1038/bjc.1952.30

**Published:** 1952-09

**Authors:** M. A. Epstein


### THE EFFECT ON THE ROUS NO. 1 FOWL SARCOMA VIRUS OF BERKEFELD FILTRATION WITH AND WITHOUT PREVIOUS REPEATED FREEZING AND THAWING.*

M. A. EPSTEIN.

*From the Bland-Sutton Institute of Pathology, Middlesex Hospital, London, W.1.*

Received for publication July 17, 1952.

It has been reported in the past that Berkefeld filtrates made from Rous No. 1 fowl sarcoma material which had been subjected to repeated applications of cold possessed a greater tumour-producing activity than those prepared from similar material which had not been subjected to this treatment (Selbie and McIntosh, 1939). In explanation of this finding Selbie and McIntosh (1939) suggested that the freezing broke up the tissue fragments, and thus allowed a greater liberation of virus than would otherwise have been possible.

In the course of some recent quantitative investigations into the effects of cold and desiccation on the Rous No. 1 fowl sarcoma (Epstein, 1951) it was found that when Rous tumour material was repeatedly frozen and thawed and then extracted, no such greater liberation of virus took place than where no treatment was applied. Virus suspensions made from the treated material had less tumour-producing activity than those made from similar untreated samples ; thus the suggestion made by Selbie and McIntosh (1939) was not confirmed.

However, although no increased liberation of virus from Rous material subjected to repeated freezing and thawing could be demonstrated to account for the increased tumour-producing activity of Berkefeld filtrates made from such material reported by Selbie and McIntosh (1939), there remained another possible explanation of their finding. The increased tumour-producing activity of Berkefeld filtrates prepared from Rous tumour subjected to repeated applications of cold might have been due to some action of the cold on the virus whereby its physical state was altered in such a way as to allow it to pass the filter with greater facility than where no treatment was applied. It was therefore decided to investigate the effect of repeated applications of cold on the filterability of the Rous virus as distinct from its extractability.

As a preliminary to this it was considered necessary to examine the effect of Berkefeld filtration on suspensions of the Rous virus. For although it has long been known that the procedure may render highly active Rous virus preparations inert (Rous and Murphy, 1914), and although this has, since it was first reported, often been taken for granted (Cramer and Foulds, 1930), few experimental investigations of the phenomenon have been undertaken. That particles in suspension became adsorbed on to Berkefeld filters during filtration has been known for some time and the influence of pH upon this has been demonstrated (Mudd, 1922 ;

* This communication formed one part of a thesis approved by the University of London for the degree of Ph.D.

Kramer, 1927). However, no work of this kind has been reported with regard to the Rous virus. The chromatographic study of Riley (1950) in which adsorption of the Rous virus was shown to take place on to a column of diatomaceous earth suggested that adsorption would also take place when this virus was passed through Berkefeld filters which are likewise composed of diatomaceous earth.

In the present work an attempt has been made to assess the decrease in the tumour-producing activity of suspensions of the Rous No. 1 fowl sarcoma virus which Berkefeld filtration brings about. Experiments are also reported which were designed to show whether or not this decrease was due to adsorption of the virus on to the Berkefeld candle during filtration, and in addition, an investigation has been carried out to find whether repeated freezing and thawing affects the filterability of the Rous virus. A titration technique has been used.

### MATERIALS AND METHODS.

*Tumour.*—The Rous No. 1 fowl sarcoma used was derived from that introduced into the Bland-Sutton Institute in 1925, and maintained by passage since then.

*Animals.*—Pedigreed Brown Leghorn fowls from the Institute of Animal Genetics, Edinburgh, have been used in the experiments. They were between 7 and 17 weeks old when inoculated, and belonged to three susceptible inbred lines developed at Edinburgh, or crosses derived from them. The lines and crosses used were Intensity, Breeding, Non-moult, Breeding hen × Intensity cock, Breeding hen × Non-moult cock, and Intensity hen × Breeding cock, and have been described by Greenwood, Blyth and Carr (1948) ; both the age at which the birds were inoculated and the line or cross to which they belonged were determined by the exigencies of supply.

### *Preparation of tumour material for the experiments.*

For each experiment a bird with a large actively growing sarcoma of anything from 9 to 18 days' growth from the date of inoculation was selected as a source of tumour material and killed by wringing of the neck. The tumour was removed, cut small with scissors, and disintegrated in a mechanical blender ; the method has been described in detail in a previous paper (Epstein and Cook, 1951). When prepared, the disintegrated tumour was loaded into a Record syringe for easy manipulation.

### *Techniques used in the experiments.*

*Repeated freezing and thawing.*—Where disintegrated tumour was to be treated in this way it was loaded into ampoules from a Record syringe, shell-frozen, and at once thawed. The process of freezing and thawing was rapidly repeated five times, after which the material was ready for use. A detailed account of the method has previously been given elsewhere (Epstein, 1951).

*Berkefeld filtration.*—Material to be filtered in this way was passed through a Berkefeld V candle (German laboratory type 12, $2\frac{1}{2} \times \frac{5}{8}$ in., supplied by Baird & Tatlock, Ltd., London) using a negative pressure of 380–510 mm. Hg. obtained by means of a water pump. Filtration was continued until the filter candle was quite empty, as shown by air bubbles being sucked through after the filtered fluid. After use each filter candle was tested by passing through it 80 c.c. of an overnight broth culture of *Staph. aureus* and *Chr. prodigiosum* diluted 1 in 16 in broth. Of the filtrate obtained in this way 1 c.c. was incubated in a tube of broth at 37° C. for 18 hours and was examined after that period for the presence of the test organisms. The results of these tests are shown with the corresponding experimental results in Tables I, II and III.

*Preparations used for inoculation.*

All samples of disintegrated tumour, both those left untreated and those subjected to repeated freezing and thawing, were made in each experiment into some or all of the following preparations :

*Virus suspension.*—This consisted of a 10 per cent. vol./vol. suspension of disintegrated tumour in physiological saline from which the cells were separated off by centrifuging in an angle centrifuge at 3500 r.p.m. for 15 minutes, decanting the supernatant fluid obtained, and centrifuging it further in an horizontal centrifuge at 6000 r.p.m. for another 15 minutes. The resulting clear supernatant fluid was decanted and used here as the virus suspension.

*Berkefeld filtrate.*—This was prepared by passing virus suspension through a Berkefeld filter in the manner already described above.

*Saline wash.*—Physiological saline was passed through a Berkefeld filter after it had been used and was collected in a fresh unused filter flask ; the volume of the saline was kept the same as that of the original material for which the filter had been used.

*Experimental procedure.*

*Experiments* 1, 2 *and* 3 (effect of filtration).—Disintegrated Rous tumour was prepared and from it a virus suspension was made as has been described. A 1 c.c. sample of the virus suspension was taken and diluted in serial tenfold steps with physiological saline, a portion of each dilution being kept ready for inoculation ; the rest of the virus suspension was passed through a Berkefeld filter. A sample was taken from the resulting filtrate, part of it being set aside ready for inoculation and part being used to make dilutions in serial tenfold steps with saline ; a portion of each dilution was kept for inoculation.

*Experiments* 4 *and* 5 (recovery of virus from filter).—These experiments were performed in their early part as a repetition of the foregoing in which samples of a virus suspension and the Berkefeld filtrate derived from it were collected and diluted in serial tenfold steps with saline for inoculation. In addition, however, a saline wash of the filter was prepared ; a sample was taken from the saline wash, part being set aside undiluted for inoculation and part being used to make dilutions in serial tenfold steps with saline. A portion of each dilution was kept for inoculation.

*Experiments* 6, 7, 8 *and* 9 (effect of freezing and thawing on filterability).—Disintegrated tumour was prepared and in each of these experiments was divided into two samples, one being left untreated and the other being subjected to repeated freezing and thawing. The two samples of disintegrated tumour, that left untreated and that subjected to repeated freezing and thawing, were each made into a virus suspension and a Berkefeld filtrate ; all of these preparations were diluted in serial tenfold steps with saline ready for inoculation.

*Inoculations.*—Inoculations were made into the breast and thigh muscles of fowl; each inoculum was 0·5 c.c. in volume, and four inoculations of each preparation or dilution of a preparation were made. The scheme used for the distribution of the inoculations amongst the birds has been described elsewhere (Epstein, 1951).

*Examination of fowl.*—All the birds were examined every week, and all the tumours which developed, including those few which subsequently regressed, were recorded. Every bird which died or which was killed at the conclusion of an experiment was submitted to *post-mortem* examination; histological investigations were carried out upon all material the nature of which could not be diagnosed macroscopically.

*Duration of experiments.*—The experiments were continued until all the tumour-bearing birds had died, or for a minimum period of nine weeks, after which time the surviving birds were killed.

*General considerations.*

Inoculations were taken as having caused a tumour in all cases where tumours were palpated at the site of inoculation. or where a tumour was found there at *post-mortem* examination.

Strict aseptic technique was maintained throughout all the experimental procedures. All the experimental procedures were timed. None was of more than 4½ hours' duration up to the moment of completing the inoculations and thus the time in which gradual inactivation of the virus by oxidation (Pirie and Holmes, 1931) could take place was standardised.

RESULTS.

Table I shows that the tumour-producing activity of a Berkefeld filtrate of the Rous sarcoma was in each case less than that of the virus suspension from which it was made; the reduction in this activity which followed the filtration was in one case very considerable (Experiment 3).

TABLE I.—*The Effect of Berkefeld Filtration on the Tumour-producing Activity of Preparations of the Rous Virus.*

| Expt. No. | Preparation of disintegrated tumour. | Dilutions of preparation and number of tumours arising from 4 inoculations of each. | | | | | | Filter test. | |
|---|---|---|---|---|---|---|---|---|---|
| | | $10^{-0}$ | $10^{-1}$ | $10^{-2}$ | $10^{-3}$ | $10^{-4}$ | $10^{-5}$ | *prod.* | *Staph.* |
| 1 | 10% virus suspension | – | – | 3 | 3 | 0 | 0 | – | – |
| | Berkefeld filtrate | 4 | 4 | 3 | 0 | – | – | R | R |
| 2 | 10% virus suspension | – | – | 4 | 4 | 1 | 0 | – | – |
| | Berkefeld filtrate | 4 | 4 | 3 | 0 | – | – | P | R |
| 3 | 10% virus suspension | – | 4 | 4 | 2 | 1 | 0 | – | – |
| | Berkefeld filtrate | 1+(1) | 0 | 0 | 0 | 0 | – | R | R |

Tumours which regressed shown in brackets.
P = passed. R = retained.

It will be seen from Table II that where physiological saline was passed through a Berkefeld candle immediately after the latter had been used to filter a Rous sarcoma virus suspension, the saline acquired a considerable tumour-producing activity. In addition, the results shown in Table II confirm those of Table I regarding the reduction in the tumour-producing activity of Rous sarcoma virus suspensions which takes place when these are passed through a Berkefeld filter candle.

TABLE II.—*The Tumour-producing Activity of Saline Passed through Berkefeld Candles Previously used to Filter Preparations of the Rous Virus.*

| Expt. No. | Preparation of disintegrated tumour. | Dilutions of preparation and number of tumours arising from 4 inoculations of each. | | | | | | Filter test. | |
|---|---|---|---|---|---|---|---|---|---|
| | | $10^{-0}$ | $10^{-1}$ | $10^{-2}$ | $10^{-3}$ | $10^{-4}$ | $10^{-5}$ | *prod.* | *Staph.* |
| 4 | 10% virus suspension | – | 4 | 4 | 0 | 0 | 0 | – | – |
| | Berkefeld filtrate | – | 4 | 1 | 0 | 0 | – | R | R |
| | Saline wash | 4 | 2 | 1 | 0 | – | – | – | – |
| 5 | 10% virus suspension | – | 4 | 4 | 4 | 1 | 0 | – | – |
| | Berkefeld filtrate | – | 3 | 1 | 0 | 0 | 0 | R | R |
| | Saline wash | 3 | 3 | 2 | 0 | 0 | – | – | – |

Tumours which regressed shown in ( ).
P = passed. R = retained.

From Table III it is evident that the tumour-producing activity of virus suspensions made from disintegrated Rous tumour which had been subjected to

TABLE III.—*The Effect of Repeated Freezing and Thawing on the Tumour-producing Activity of Preparations of the Rous Virus and on their Filterability*

| Expt. No. | State of disintegrated tumour. | Preparation. | Dilutions of preparation and number of tumours arising from 4 inoculations of each. | | | | | | Filter test. | |
|---|---|---|---|---|---|---|---|---|---|---|
| | | | $10^{-0}$ | $10^{-1}$ | $10^{-2}$ | $10^{-3}$ | $10^{-4}$ | $10^{-5}$ | *prod.* | *Staph.* |
| 6 | Untreated | 10% virus suspension | – | 4 | 4 | 4 | 2 | 0 | – | – |
| | Frozen and thawed × 6 | 10% virus suspension | – | 4 | 4 | 3 | 0 | 0 | – | – |
| | Untreated | Berkefeld filtrate | 3 | 2 | 0 | 0 | 0 | – | R | R |
| | Frozen and thawed × 6 | Berkefeld filtrate | 4 | 0 | 0 | 0 | 0 | – | P | R |
| 7 | Untreated | 10% virus suspension | – | 4 | 4 | 4 | 3 | 0 | – | – |
| | Frozen and thawed × 6 | 10% virus suspension | – | 4 | 4 | 3 | 0 | 0 | – | – |
| | Untreated | Berkefeld filtrate | 4 | 2 | 0 | 0 | 0 | – | C | C |
| | Frozen and thawed × 6 | Berkefeld filtrate | 2+(2) | 0 | 0 | 0 | 0 | – | R | R |
| 8 | Untreated | 10% virus suspension | – | 4 | 4 | 4 | 3 | 0 | – | – |
| | Frozen and thawed × 6 | 10% virus suspension | – | 4 | 4 | 4 | 0 | 0 | – | – |
| | Untreated | Berkefeld filtrate | 3 | 4 | 2 | 0 | 0 | – | P | R |
| | Frozen and thawed × 6 | Berkefeld filtrate | 4 | 4 | 0 | 0 | 0 | – | R | R |
| 9 | Untreated | 10% virus suspension | – | 4 | 3 | 3 | 0 | 0 | – | – |
| | Frozen and thawed × 6 | 10% virus suspension | – | 3+(1) | 3 | 3 | 0 | 0 | – | – |
| | Untreated | Berkefeld filtrate | 4 | 4 | 2 | 0 | 0 | – | R | R |
| | Frozen and thawed × 6 | Berkefeld filtrate | 4 | 4 | 0 | 0 | 0 | – | R | R |

Tumours which regressed shown in ( ).
P = passed. R = retained. C = test contaminated.

repeated freezing and thawing was usually reduced (Experiments 6, 7 and 8) as compared with those made from untreated material. Where the activity of the preparation made from frozen and thawed disintegrated tumour was not less than that made from untreated material it was the same (Experiment 9), not greater. Table III also shows that the same order of reduction in tumour-producing activity took place when a virus suspension made from untreated disintegrated tumour was passed through a Berkefeld candle to give a filtrate as when a virus suspension made from repeatedly frozen and thawed material was so filtered. That such a reduction took place confirms the results of Table I.

Also shown in Tables I, II and III are the results of the tests made with each filter after use to determine its power of retaining the organisms of the test cultures. On several occasions the filters failed to retain *Chr. prodigiosum* (Table I, Experiment 2; Table III, Experiments 6 and 8), the smaller of the two test organisms. In one experiment (Table III, Experiment 7) the test was inconclusive owing to accidental contamination.

### DISCUSSION.

The results that have been obtained show that where an active virus suspension in physiological saline, prepared from disintegrated Rous tumour, was passed through a Berkefeld V candle, the tumour-producing activity of the resulting filtrate was always less than that of the original virus suspension (Tables I, II and III).

The tumour-producing activity of the virus suspensions is considered to have been due solely to the virus they contained; such suspensions are believed to be largely if not wholly cell-free for reasons which have been considered at length elsewhere (Epstein, 1951). Now, from the results shown in Table II, it is clear that an appreciable amount of the tumour-producing activity lost when virus suspensions were passed through Berkefeld candles could be recovered by washing saline through the latter after use. In the experiments shown in Table II the filters when tested retained in each case both *Chr. prodigiosum* and *Staph. aureus* making it impossible for the lost tumour-producing activity, recovered on washing the filters with saline, to have been due to cells. This recoverability of at least part of the tumour-producing activity lost on filtering Rous virus suspensions gives further support for believing the latter to owe their activity almost wholly to virus.

The reduction in virus content, often very considerable (Table I, Experiment 3), brought about by Berkefeld filtration of Rous virus suspensions appears to be due to adsorption of the virus on to the filter rather than to it being held up by the filter on account of its size. This is considered to be the case since the lost virus was recovered at least in part when saline was washed through the filter (Table II), and since the failure of the filters on three occasions to hold back one of the organisms with which they were tested after use did not affect the amount of virus which came through them as judged by the tumour-producing activity of the filtrates (Tables I and III, Experiments 2, 6 and 8). This view is supported by the chromatographic studies of Riley (1950), in which the Rous virus was adsorbed on to columns of diatomaceous earth—the same material as that of which Berkefeld filters are compased—and was eluted off them by washing with saline, and by work on the adsorption on to Berkefeld filters during filtration of particles other than viruses (Mudd, 1922; Kramer, 1927).

The results of the concluding experiments of the present work (Table III) show that the same order of reduction in tumour-producing activity took place where virus suspensions made from disintegrated tumour treated by repeated freezing and thawing were passed through a Berkefeld candle to give a filtrate, as where those made from similar untreated disintegrated tumour were so filtered. The application of repeated freezing and thawing to Rous sarcoma material has thus been found not to increase the ability of the virus contained in it to pass Berkefeld filters. The present work has also shown (Table III), in confirmation of that previously reported (Epstein, 1951), that repeated freezing and thawing of mechanically disintegrated Rous tumour material did not release more virus on extraction than where no such treatment was applied. From these two facts it appears that repeated freezing and thawing has no action on the Rous virus likely to make Berkefeld filtrates of treated tumour material, prepared as described here, more active than those made from untreated material. The results shown in Table III demonstrate this clearly ; they are at variance with those reported by Selbie and McIntosh (1939).

In attempting to relate the results of the present work to those of previous investigators with which they do not agree, two possible explanations require consideration :

Firstly, it must be remembered that Selbie and McIntosh carried out their investigations 13 years ago. During that time the Rous virus may possibly have undergone variations affecting its properties which might account for the variations in the experimental results obtained.

Secondly, the method of extracting the Rous virus employed by Selbie and McIntosh (1939) consisted of coarse mincing of the tumour material followed by grinding with sand. Repeated freezing and thawing was then applied to the resulting preparation, and may well have contributed to the achieving of complete disintegration of the tumour and the maximum extraction of virus from it. In the present work, however, disintegration of the tumour material was brought about mechanically before the cold was applied, and would seem to have been so complete that the repeated freezing and thawing could not have any further effect. This is considered to be the most likely explanation of the contradiction between the findings reported by Selbie and McIntosh (1939) and those recorded in this communication.

In consequence of the results of the present work it is thought that where in the future attempts are to be made to demonstrate the presence of viruses in tumours not hitherto known to possess them, Berkefeld filtration, with or without previous repeated freezing and thawing of the material to be filtered, should be avoided. Tumour extracts freed from cells by centrifugation would be more suitable preparations for such work than filtered extracts, being more likely to retain small quantities of a virus than preparations which had been passed through Berkefeld filters with their property of adsorption of virus particles.

### CONCLUSIONS.

The present work has shown that the Rous virus becomes adsorbed on to Berkefeld filters during filtration, and that it can be washed out of them, when so adsorbed, with saline. The extent of the loss of virus which always takes place during Berkefeld filtration owing to this adsorption has been demonstrated by the titration methods used, and has been shown, on occasions, to be very great. The alleged increase in tumour-producing activity of Berkefeld filtrates made from Rous sarcoma material subjected to repeated freezing and thawing, as compared with those made from untreated material, has been investigated, and has not been substantiated with the methods used here. It has been confirmed that repeated freezing and thawing of mechanically disintegrated Rous sarcoma tissue prior to extraction does not cause the liberation of more virus from the cells than takes place when no treatment is applied, and at the same time it has been demonstrated that the process of repeated freezing and thawing does not increase the ability of the Rous virus to pass Berkfeeld filter candles.

From the foregoing it has been concluded that Berkefeld filtration of tumour extracts with or without prior repeated freezing and thawing is not a suitable technique for use in work designed to demonstrate a virus in tumours not as yet known to possess one.

### SUMMARY.

Experiments performed to establish the effect of Berkefeld filtration on suspensions of the Rous No. 1 fowl sarcoma virus are described. Also described are experiments in which the effect of repeated freezing and thawing on the filterability of this virus has been investigated. Susceptible inbred Brown Leghorn fowl have been used.

The method used to disintegrate Rous sarcoma tissue mechanically in order to make from it a virus suspension is set forth. The technique used to apply repeated freezing and thawing to disintegrated Rous tumour is indicated, as is the technique used in Berkefeld filtration of suspensions of the virus.

A titration method has been employed in which preparations whose tumour-producing activities were to be compared have been diluted in serial tenfold steps with saline and inoculations made with each dilution.

The results show that when Rous virus suspensions prepared by extracting disintegrated Rous tumour with saline were passed through Berkefeld filters, part of the virus became adsorbed on to the filter. By washing saline through the filters immediately after use much of the adsorbed virus proved recoverable.

The results have confirmed that repeated freezing and thawing of mechanically disintegrated Rous tumour before preparing extracts from it did not allow a greater liberation of the virus than would otherwise have occurred.

It has also been demonstrated that repeated freezing and thawing of disintegrated Rous sarcoma material did not increase the ability of the Rous virus it contained to pass Berkefeld filters when extracted and suspended in saline.

The results are discussed in detail together with the conclusions drawn from them.

The expenses of this investigation were borne by the British Empire Cancer Campaign.

### REFERENCES.

Cramer, W., and Foulds, L.—(1930) *Sci. Rep. Cancer Res. Fd., London*, **9**, 33.
Epstein, M. A.—(1951) *Brit. J. Cancer*, **5**, 317.
*Idem* and Cook, H. F.—(1951) *Ibid.*, **5**, 244.
Greenwood, A. W., Blyth, J. S. S., and Carr, J. G.—(1948) *Ibid.*, **2**, 135.
Kramer, S. P.—(1927) *J. infect. Dis.*, **40**, 343.
Mudd, S.—(1922) *Amer. J. Physiol.*, **63**, 429.
Pirie, Antoinette, and Holmes, Barbara E.—(1931) *Brit. J. exp. Path.*, **12**, 127.
Riley, V.—(1950) *J. nat. Cancer Inst.*, **11**, 199.
Rous, P., and Murphy, J. B.—(1914) *J. exp. Med.*, **19**, 52.
Selbie, F. R., and McIntosh, J.—(1939) *Brit. J. exp. Path.*, **20**, 443.